# Bridging the Continuous Glucose Monitoring decision gap: from glycaemic variability data to actionable stability in diabetes care

**DOI:** 10.3389/fendo.2026.1862387

**Published:** 2026-05-29

**Authors:** Qingmei Wang, Fang Pan, Bowu Li, Xueen Liu, Jiale Zhang

**Affiliations:** 1 Department of Nursing, Beijing Hepingli Hospital, Beijing, China; 2 Beijing Hepingli Hospital, Beijing, China; 3 Department of Endocrinology, Beijing Hepingli Hospital, Beijing, China; 4 China Science and Technology Development Center for Chinese Medicine, Beijing, China

**Keywords:** continuous glucose monitoring, glycaemic variability, precision medicine, time in tight range, type 2 diabetes

## Abstract

The 2026 proposal to stage type 2 diabetes by β-cell trajectory and continuous glucose monitoring (CGM) metrics—specifically time in tight range (TITR)—represents a pivotal advance in dysglycaemia phenotyping. Yet, this innovation exposes a critical translational void: actionable algorithms to convert granular CGM outputs into stage-specific, stability-oriented therapeutic responses remain absent from clinical guidelines. This Perspective identifies three barriers perpetuating this decision gap: (i) metric overload in ambulatory glucose profiles, unaccompanied by decision-support pathways linking variability and TITR decline to therapeutic escalation; (ii) clinical inertia that prioritizes hypoglycaemia avoidance (TBR) over stability optimization (TITR, CV reduction), despite compelling evidence associating glycaemic variability with cardiovascular sequelae; and (iii) a conspicuous absence of structured frameworks for embedding CGM-derived behavioural insights into sustainable lifestyle routines. To address these impediments, a pragmatic, three-step closed-loop clinical model is proposed, stratifying CGM interpretation and action thresholds according to the new disease stages (1–3b). Central to this framework is the prioritization of sustainable glycaemic stability—operationally defined by a coefficient of variation ≤36%, stage-appropriate TITR attainment, and time below range <4%. The model offers provisional templates for stage-concordant lifestyle prescription and pharmacotherapy coordination, while acknowledging the risk of overtreatment, the importance of patient-reported outcomes, and the substantial implementation barriers that constrain real-world adoption. Bridging the CGM decision gap demands prospective validation of stage-specific targets and the seamless integration of context-aware decision-support tools into electronic health records.

## Introduction

**Figure 1:**
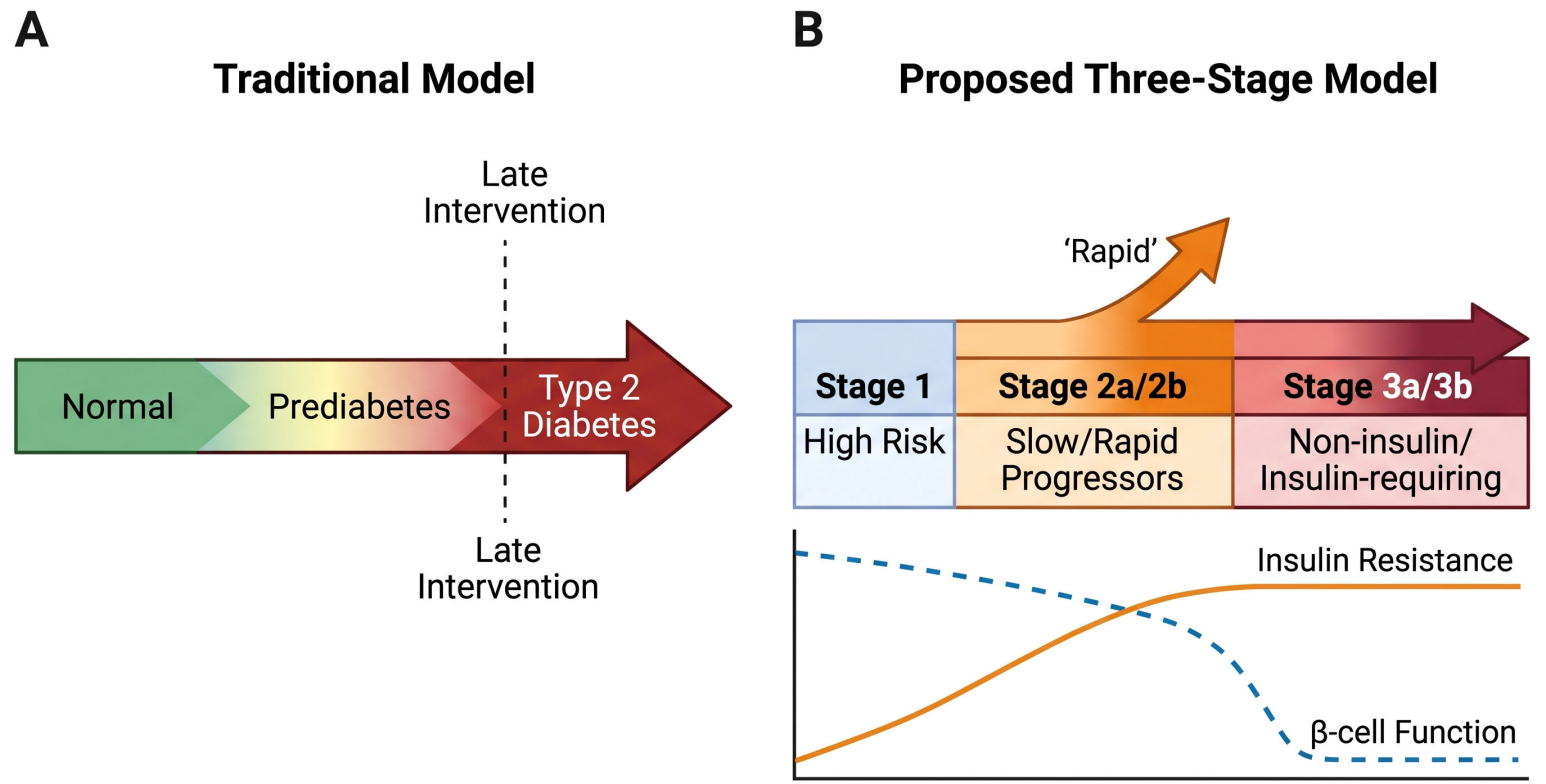
Stage-based reclassification of type 2 diabetes replacing the traditional “prediabetes” construct. **(A)** The traditional paradigm depicts a linear continuum from normal glucose tolerance through an ill-defined “prediabetes” state to type 2 diabetes, with intervention typically deferred until diagnostic thresholds are crossed. **(B)** The proposed three−stage model grounds classification in progressive β−cell dysfunction. Stage 1 (high risk) individuals maintain normoglycaemia (fasting plasma glucose <5.6 mmol/L) through compensatory insulin secretion. Stage 2 (dysglycaemia) is stratified into slow progressors (2a; older onset, milder hyperglycaemia) and rapid progressors (2b; younger onset, obesity, 1−h OGTT glucose ≥8.6 mmol/L, declining C−peptide). Stage 3 (clinical diabetes, ~70–80% β−cell loss) is subdivided into 3a (non−insulin agents) and 3b (insulin−requiring). Time in tight range (TITR; 3.9–7.8 mmol/L) serves as an adjunct staging metric, with approximate thresholds of 90–95% (Stage 1), 80–90% (Stage 2), 70–85% (Stage 3a), and 60–75% (Stage 3b). All stage−specific TITR thresholds are provisional estimates requiring prospective validation. The schematic also depicts the inverse trajectories of β−cell mass (declining) and insulin resistance (rising then plateauing), underscoring the pathophysiological rationale for reclassification. TITR, time in tight range; OGTT, oral glucose tolerance test.

The limitations of current diagnostic criteria for type 2 diabetes—based on glucose thresholds and glycated haemoglobin—are increasingly apparent, as they often identify individuals at risk too late to enable optimal preventive intervention (1). In January 2026, a Lancet Diabetes & Endocrinology commentary proposed abandoning the term “prediabetes” in favour of a three-stage model of type 2 diabetes grounded in progressive β-cell function decline and glycaemic trajectory (2). Stage 1 includes individuals at elevated risk of dysglycaemia, defined by validated country-specific risk scores, with normal glucose levels (fasting plasma glucose <5.6 mmol/L) despite gradual minimal β-cell loss compensated by increased insulin secretion. Stage 2 (dysglycaemia) is subdivided into 2a (slow progressors, typically diagnosed after age 65 years with milder hyperglycaemia) and 2b (rapid progressors: younger onset, obesity, 1-h OGTT glucose ≥8.6 mmol/L despite normal 2-h values, and low C-peptide). Stage 3 (clinical diabetes) reflects approximately 70–80% β-cell loss and is divided into 3a (initially manageable with non-insulin agents) and 3b (requiring insulin initiation). The framework introduces TITR (time spent in 3.9–7.8 mmol/L) as an adjunct staging metric, with provisional thresholds of 90–95% in Stage 1, 80–90% in Stage 2, and <80% in Stage 3. It must be emphasised that all stage−specific TITR thresholds are provisional estimates. The proposed TITR threshold for Stage 1 is derived from physiological reasoning rather than empirical data; in true normoglycaemic individuals, TITR is expected to approach 100%, and values <95% should be regarded as an early warning signal of incipient dysglycaemia. **Figure 1** provides a visual comparison of the traditional “prediabetes” construct with the proposed three−stage model, and maps the corresponding TITR thresholds alongside the trajectories of β−cell mass and insulin resistance across the disease continuum.

This reclassification is more than semantic. It reframes early dysglycaemia as a treatable disease state rather than a reversible “pre-” condition, counters therapeutic inertia, eliminates false reassurance for patients and clinicians, and creates a regulatory pathway for preventive pharmacotherapy in Stage 2 (2). Recent analysis of the ELSA-Brasil cohort validated a staging schema based on 1-hour post-load plasma glucose, demonstrating progressive β-cell compensation decline (ISSI-2) across stages and confirming that adding 1-h PG testing doubled detection of unknown diabetes—providing empirical support for the staging approach (note: the staging criteria in this study, based on 1-h PG ≥155 mg/dL and ADA/WHO-IEC definitions, differ slightly from the generalised model described above) (3). Phenotypic precision further refines decision-making: intensive lifestyle and early pharmacologic intervention for rapid progressors (Stage 2b) versus conservative management to avoid overtreatment in slow progressors (Stage 2a) (2).

CGM metrics are thereby elevated from management tools to components of disease staging and early warning. The 2026 ADA Standards of Care reinforce this momentum by endorsing CGM at diabetes onset and for any treatment regimen where it can aid management, with core targets of TIR >70%, TBR <4%, and CV ≤36% (4, 5). The 2025 Japanese consensus statement provides detailed guidance on interpreting and responding to TIR, TAR, and TBR readings for both type 1 and type 2 diabetes, emphasising that the overarching principle is to achieve TIR targets by decreasing TAR while minimising TBR to avoid hypoglycaemia (6). The Latin American expert consensus further provides a standardised methodology for CGM data interpretation, establishing that reliable interpretation requires at least 70% sensor use over 14 days, with the ambulatory glucose profile (AGP) serving as the primary tool; TITR was considered but not formally included due to limited supporting evidence and regional technology access barriers (7).

An important distinction must be drawn between conventional TIR (3.9–10.0 mmol/L) and TITR (3.9–7.8 mmol/L) (8). Standard TIR captures overall euglycaemia but includes glucose values up to 10.0 mmol/L—a range that already encompasses postprandial hyperglycaemic excursions and subclinical dysglycaemia. TITR, by narrowing the upper limit to 7.8 mmol/L, specifically excludes this borderline hyperglycaemic zone. Consequently, TITR functions as a more stringent metric of physiological stability and a more sensitive early warning indicator of β−cell decompensation. Emerging evidence suggests that TITR is independently associated with cardiovascular and all−cause mortality, whereas standard TIR may be less discriminatory in early−stage disease (9). Thus, while TIR remains a pragmatic goal for established diabetes, TITR may offer incremental value for early detection and stability−focused management.

Yet a decision-translation gap persists. Guidelines define what constitutes good control but provide limited instruction on how to respond when TITR declines, CV exceeds 36%, or patterns reveal lifestyle-specific excursions. CGM does not replace HbA1c; the glucose management indicator (GMI) serves only as an adjunct, particularly when discordance exceeds 0.5%, prompting investigation for non-glycaemic confounders such as haemoglobinopathy, anaemia, or chronic kidney disease (4). CGM’s unique contribution is the capture of dynamic glycaemic stability—quantified by CV and TITR—which is independently associated with oxidative stress, endothelial dysfunction, and cardiovascular risk beyond mean glucose (10, 11). A systematic review of CGM metrics and microvascular complications found that lower TIR was consistently associated with composite microvascular complications and hospitalisation for hypoglycaemia or ketoacidosis, while CV ≥36% was linked to higher likelihood of hypoglycaemia and glucose instability (12). The therapeutic objective must therefore be sustainable stability (CV ≤36%, high TITR/TIR, minimal TBR) coupled with the cultivation of replicable healthy behaviours, with hypoglycaemia avoidance as a foundational safety boundary rather than the sole endpoint. **Box 1** provides operational definitions of glycaemic variability, glycaemic stability, sustainable stability, and stability optimization as used in this framework.

**Box 1. Key Terminology: Glycaemic Stability Framework**

**Glycaemic variability (GV)**. The raw phenomenon of glucose fluctuations over time, objectively quantified by the coefficient of variation (CV). High GV reflects erratic glucose excursions and is independently associated with oxidative stress and cardiovascular risk.**Glycaemic stability**. The desired physiological state of low glycaemic variability, operationally defined as a coefficient of variation (CV) ≤36%. It represents a quantifiable treatment target but does not inherently account for the sustainability or behavioural origins of that stability.**Sustainable stability**. The overarching treatment paradigm proposed in this Perspective. Sustainable stability extends the metric-based concept of glycaemic stability by integrating (i) stage-appropriate time in tight range (TITR) attainment, (ii) minimal time below range (TBR <4%), and (iii) crucially, achievement through replicable, patient-centred behaviours that minimise treatment burden, alarm fatigue, and psychological burnout.**Stability optimization**. The iterative clinical process of titrating pharmacotherapy and structuring lifestyle interventions to move a patient from a state of glycaemic variability towards sustainable stability. It requires balancing TITR improvement, CV reduction, and hypoglycaemia avoidance while respecting individual preferences, frailty, and quality of life.

## The staging innovation and its clinical imperative

The three-stage model directly addresses longstanding limitations of the prediabetes construct. It acknowledges dysglycaemia as an independent driver of cardiovascular disease, chronic kidney disease, and selected malignancies, not merely a precursor (2). It counters clinical inertia by labelling early dysglycaemia as a diagnosable and actionable disease state. Most importantly, it paves the way for regulatory approval of therapies that delay progression, previously hindered by the absence of formal guidance for “prediabetes.”

TITR adds granular sensitivity. Defined as percentage time in 3.9–7.8 mmol/L, it detects subtle excursions from normoglycaemia that precede conventional diagnostic thresholds (13). The clinical relevance of TITR is increasingly supported by prospective data: a 2025 prospective cohort study demonstrated that lower TITR is independently associated with increased risk of all-cause and cardiovascular mortality in patients with type 2 diabetes, suggesting that tight glycaemic control within the physiological range may be crucial for reducing long-term mortality risk (9). Real-world evidence further shows TITR improves with adjunctive therapies such as oral semaglutide and aligns with treatment modality in hybrid closed-loop systems (14). Additionally, glycated albumin at a threshold of 17.4% has been shown to effectively identify type 2 diabetes patients with TITR >50%, providing a complementary laboratory marker for settings where CGM access is limited (15).

Emerging evidence suggests that the integration of CGM with artificial intelligence may support more precise identification of at-risk individuals and personalised interventions (16). Similarly, CGM offers additional insights into glycaemic dynamics beyond traditional markers, potentially aiding early detection and patient engagement in high-risk states (17). The staging advance therefore generates a new clinical imperative: metrics must be translated into management decisions that differ meaningfully across stages. Without such pathways, the promise of earlier intervention and phenotypic precision remains unrealised, and patients continue to experience glycaemic excursions that are visualised but not durably stabilised.

## Three barriers perpetuating the CGM decision gap

### Barrier one: metric overload without actionable algorithms

Ambulatory glucose profile (AGP) reports now synthesise a wealth of data—median glucose curve, interquartile and interdecile ranges, TIR, TAR, TBR, GMI, and CV—yet most outpatient encounters reduce interpretation to visual colour-band inspection. For a patient in Stage 2b presenting with TITR <85% and CV >36%, the clinician observes postprandial excursions but lacks a systematic map from these deviations to concrete next-step actions: intensified lifestyle counselling, early pharmacotherapy, or both. Non-glycaemic predictors of elevated CV (e.g., sulfonylurea or prandial insulin use conferring odds ratios of 4.7–5.2; each 5-year increment in diabetes duration or 10 mL/min/1.73 m² decrement in eGFR increasing odds by ~20%) are identifiable yet remain untranslated into stage-tailored algorithms (18). The consequence is missed opportunity: rapid progressors—who stand to gain most from early stabilisation—frequently progress without timely, data-driven intervention.

### Barrier two: stability prioritisation versus hypoglycaemia avoidance

Clinicians, appropriately vigilant about TBR, frequently sacrifice TITR and TAR reduction to maintain an ultra-low hypoglycaemia burden. This conservatism, while protective in the short term, undermines long-term stability, especially during transitions from Stage 2b to 3a or 3a to 3b. Reanalysis of DCCT data via virtual CGM traces demonstrated that hyperglycaemia-related metrics (lower TIR/TITR, higher TAR) were consistently associated with increased cardiovascular events (adjusted hazard ratios ~1.29–1.31 per standard deviation change), whereas TBR showed no increased risk and even trended protective (8). Glycaemic variability itself, independent of mean glucose, drives oxidative stress, endothelial dysfunction, and subclinical atherosclerosis (8, 9) (19). The decision gap persists because clinicians lack explicit guidance on when a modest, acceptable increase in TBR may be traded for clinically meaningful gains in TITR and overall stability.

However, it is equally critical to avoid an uncritical pursuit of TITR. Aggressive optimisation of glycaemic targets, particularly in older or frail individuals with Stage 2a or 3a disease, can inadvertently cause overtreatment, increased medication burden, alarm fatigue, and psychological distress. CGM use, while beneficial metabolically, can contribute to diabetes distress, alarm fatigue, and device burnout, particularly with frequent alerts or overly ambitious targets (20). Intensive CGM use is not uniformly beneficial; patients may experience heightened anxiety or burnout when confronted with continuous data streams. Therefore, the primum non nocere principle must serve as a fundamental boundary: stability optimisation should be pursued only when the expected reduction in long−term complications clearly outweighs the immediate burden on quality of life, treatment satisfaction, and adherence. Clinicians should individualise goals, especially in elderly populations with limited life expectancy, where symptomatic well−being and avoidance of polypharmacy take precedence over numerically defined TITR thresholds.

### Barrier three: absence of structured lifestyle integration frameworks

CGM uniquely visualises the immediate glycaemic consequences of daily behaviours: a patient can observe that consistent meal timing elevates TITR or that postprandial walking attenuates excursions. Yet no replicable framework converts these insights into “stability routines” that patients can sustain beyond the clinic visit. A 2025 randomised controlled trial illustrated the potential: prescribing 22 minutes of daily exercise timed to an individual’s CGM-identified peak hyperglycaemia significantly lowered 24-hour peak glucose and improved flow-mediated dilation compared with control, independent of HbA1c change (21).

A systematic review and meta-analysis of 21 randomised controlled trials involving 2,734 adults demonstrated that CGM-guided lifestyle interventions focusing on nutrition significantly improved HbA1c (mean difference −0.46%, 95% CI −0.71 to −0.22), TIR (mean difference +7.18%, 95% CI 2.77 to 11.58), and body weight (mean difference −2.06 kg, 95% CI −3.74 to −0.38) compared with control groups (22). Culturally tailored interventions incorporating real-time CGM have demonstrated that household members also adopt healthier lifestyles (23).

Stage−specific manifestations compound the problem. Older adults in Stage 2a risk overtreatment when isolated TITR reductions prompt unnecessary escalation; younger patients in Stage 2b exhibit poor adherence without structured, feedback−reinforced prescriptions. The absence of a personalised behavioural roadmap also undermines patient−reported outcomes: without clear links between daily choices and sustained metrics, treatment satisfaction and motivation decline, and alarm fatigue further erodes engagement. The net result is a persistent monitoring−to−action disconnect: patients see the data but lack the behavioural and emotional roadmap required for durable stability.

## A practical framework: from CGM metrics to sustainable stability

**Figure 2:**
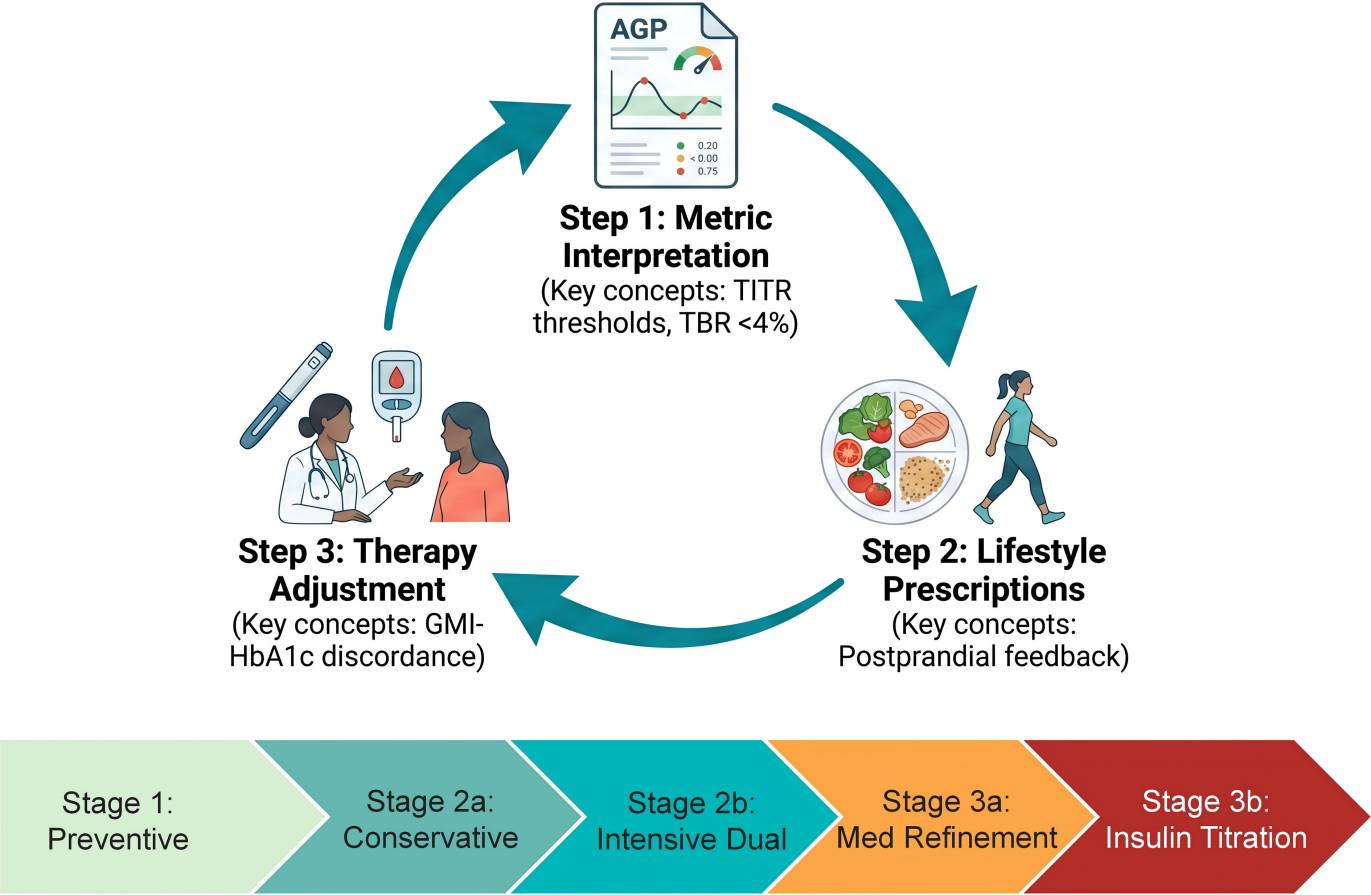
A three-step closed-loop decision framework for translating continuous glucose monitoring (CGM) metrics into stage−specific, stability−focused clinical action. The framework is organised into two tiers. The central iterative loop depicts a three-step clinical workflow: Step 1 (Metric Interpretation) verifies data adequacy against provisional thresholds—time in tight range (TITR) by stage and time below range (TBR) <4% as the safety floor; Step 2 (Lifestyle Prescriptions) structures postprandial activity, meal timing, and sleep hygiene reinforced by real-time CGM feedback; Step 3 (Therapy Adjustment) coordinates pharmacotherapy based on GMI–HbA1c discordance (>0.5%) and deterioration in stability metrics. The lower progression arrow maps intervention intensity across the five disease stages, escalating from preventive stabilisation (Stage 1), through conservative (Stage 2a) and intensive dual-modality (Stage 2b) approaches, to medication refinement (Stage 3a) and insulin algorithm-guided titration (Stage 3b). All thresholds and escalation triggers are conceptual and require prospective validation. AGP, ambulatory glucose profile; CV, coefficient of variation; GMI, glucose management indicator; TBR, time below range; TITR, time in tight range.

We propose a conceptual three-step closed-loop decision model designed to bridge metric acquisition and clinical action, explicitly tailored to the new staging system (**Table 1**). **Figure 2** depicts the operational logic of this model. The inner circular flow illustrates an iterative workflow in which the clinician (Step 1) verifies data adequacy and interprets key stability indicators against stage-specific, provisional thresholds from the AGP; (Step 2) translates identified glycaemic patterns into structured behavioural prescriptions reinforced by real-time CGM feedback; and (Step 3) adjusts pharmacotherapy guided by GMI–HbA1c discordance and stability-based triggers. The surrounding gradient arrow underscores the escalation of intervention intensity across the disease continuum—from preventive stabilisation in Stage 1, through conservative lifestyle optimisation in Stage 2a and intensive dual-modality intervention in Stage 2b, to medication refinement in Stage 3a and insulin-algorithm-guided titration in Stage 3b. The following sections detail each step.

**Table 1:** Stage-specific CGM targets and clinical action framework.

Stage	TITR range^1^	Primary stability goal	Priority clinical action	Evidence strength^2^
1 (high risk)	90–95%	CV ≤36%	Structured lifestyle; CGM feedback for adherence	TITR: physiological reasoning, not empirically validated; lifestyle: RCT evidence
2a (slow)	80–90%	CV ≤36%, TBR <4%	Conservative lifestyle; defer drugs unless TITR <80% persists	TITR: provisional estimate; action: expert consensus
2b (rapid)	80–90% (often lower)	CV ≤36%, TITR improvement	Intensive lifestyle + early pharmacotherapy (metformin ± GLP-1 RA)	Supported by indirect CV/TITR evidence and observational data
3a (non-insulin)	70–85%	CV ≤36%, TAR <25%	Medication optimisation by TIR/TITR	TITR: limited observational data; medication optimisation: guideline-aligned
3b (insulin)	60–75%	CV ≤36%, TBR <4%	Insulin titration preserving TITR; CGM-integrated algorithms	Insulin initiation trigger: conceptual, requiring validation

TITR = time in tight range (3.9–7.8 mmol/L); CV = coefficient of variation; TBR = time below range; TIR = time in range; TAR = time above range; GLP-1 RA = glucagon-like peptide-1 receptor agonist.

¹All TITR thresholds are provisional and require prospective validation in cohorts with concurrent CGM and metabolic phenotyping.

²Evidence strength reflects the basis for the recommendation: “RCT evidence” indicates support from randomised trials; “observational data” indicates support from cohort or real−world studies; “physiological reasoning” or “expert consensus” indicates hypothesis−generating concepts. None of the stage−specific TITR thresholds or escalation algorithms have been prospectively confirmed as hard endpoints.

### Step 1: rapid AGP interpretation with stage-stratified thresholds

Begin with verification of data adequacy (≥70% sensor wear over ≥14 days) (7). Confirm TBR <4% (level 2 <1%) as the non-negotiable safety floor. Prioritise TITR and CV as primary stability indicators, contextualised by stage-specific TITR ranges. When TITR falls below the stage-expected threshold and CV exceeds 36%, the immediate priority is systematic screening for modifiable lifestyle triggers (inconsistent meal timing, prolonged sedentary periods postprandially, sleep disruption) before considering pharmacologic intensification. At all stages, clinicians should evaluate the potential burden of further intervention on the patient’s quality of life and psychological well−being.

### Step 2: stage-specific lifestyle prescription templates

Translate identified patterns into structured, measurable behavioural prescriptions reinforced by CGM feedback:

Stage 1 (high risk): Preventive stabilisation via consistent meal timing, postprandial activity after the largest meal, and sleep hygiene, using real-time CGM data to reinforce adherence.

Stage 2a (slow progressors): Conservative lifestyle optimisation; reserve pharmacotherapy for persistent TITR <80% after ≥3 months of structured intervention.

Stage 2b (rapid progressors): Intensive dual-modality approach—CGM-guided prescriptions (e.g., 22-minute postprandial walk timed to peak hyperglycaemia) combined with early metformin ± GLP-1 receptor agonist.

Stage 3a (non-insulin): Shift emphasis to medication optimisation guided by TITR/TIR trends while maintaining lifestyle as foundational therapy.

Stage 3b (insulin-requiring): Insulin titration algorithms that preserve TITR stability; leverage FDA-cleared CGM-integrated basal dosing tools where available.

### Step 3: therapy coordination and structured follow-up

When GMI and laboratory HbA1c discordance exceeds 0.5%, investigate underlying non-glycaemic factors before altering therapy (4). As a concept awaiting validation, we suggest considering insulin initiation when TITR remains persistently <70% or TAR exceeds 25% despite optimised non−insulin therapy, with the explicit goal of stability rather than aggressive HbA1c reduction that risks hypoglycaemia. In Stage 3b, trigger insulin initiation by persistent TITR <70% or TAR >25% despite optimised non-insulin therapy, with the explicit goal of stability rather than aggressive HbA1c reduction that risks hypoglycaemia. Implement a tiered follow-up protocol: 4–6 weeks for patients with CV >36% or declining TITR; extend to 12 weeks once all targets are achieved. Emerging deep learning frameworks for personalised interstitial glucose prediction using CGM data may provide automated risk stratification and decision support that alleviates metric overload while preserving clinician judgment (24).

## Implementation barriers

The proposed framework is contingent on widespread CGM access and seamless clinical integration, yet substantial barriers persist. In many health systems, CGM reimbursement is restricted to individuals on intensive insulin therapy, limiting application in Stages 1–2. Even where devices are available, patients require training in sensor use and smartphone applications, while clinicians face a significant workflow burden: a full AGP interpretation may consume several minutes per patient—time rarely available in primary care. Resource−limited settings face additional constraints, including cost, internet connectivity, and shortage of trained personnel (25). To mitigate these challenges, a tiered adoption model could be considered: in basic−resource environments, priority should be given to core stability metrics (TIR, TBR, CV) with manual lifestyle counselling, while TITR−guided algorithms and EHR−integrated decision support—recently demonstrated through multivendor CGM integration—may be reserved for specialised centres (26). Equally important, patient−reported outcomes such as treatment satisfaction and diabetes distress should be systematically collected to ensure that intensifying monitoring does not inadvertently diminish quality of life.

## Clinical translation and future directions

The convergence of the three−stage classification with expanded CGM integration creates an unprecedented opportunity to reorient diabetes care from episodic, HbA1c−centric management toward continuous, stability−focused precision care. Realising this potential requires actions on three fronts.

First, guideline−developing bodies should supplement current target definitions with explicit, action−oriented implementation pathways that specify clinical triggers for reassessment, stage−stratified follow−up intervals, and standardised methods for incorporating CGM data into shared decision−making. The stage−specific framework proposed here (**Table 1**) provides a practical starting point for consensus refinement, but its recommendations should be labelled according to evidence strength to prevent premature translation into rigid mandates.

Second, EHR−embedded decision−support tools must be developed to automatically extract CGM metrics as structured data elements and generate stage−contextualised, evidence−graded recommendations at the point of care. Such tools could simultaneously flag discordant GMI–HbA1c pairs and alert clinicians to stability deterioration, thereby reducing cognitive overload. The successful multivendor CGM integration into EHRs recently demonstrated provides a template for broader implementation (26).

Third, prospective clinical studies are urgently needed to move this framework from concept to guideline−ready evidence. Specific designs could include: (a) a pragmatic randomised controlled trial in Stage 2b rapid progressors comparing a CGM−guided lifestyle−plus−early−drug arm against standard care on 2−year TITR maintenance and progression to Stage 3; (b) a factorial trial isolating the cardiovascular effect of CGM−timed exercise independent of pharmacotherapy; and (c) real−world EHR−based analyses comparing the safety and efficacy of the proposed TITR−based therapy escalation trigger (TITR <70%) against conventional HbA1c−based triggers. All such studies should incorporate patient−reported outcomes—including quality of life, diabetes distress, and treatment satisfaction—as co−primary endpoints to ensure that the pursuit of metabolic stability aligns with what matters to patients. Dedicated funding and multi−centre collaboration will be essential to generate the necessary evidence.

## Limitations

This perspective article has several important limitations. First, the stage-specific TITR thresholds and the associated therapeutic algorithms are largely derived from physiological reasoning, cross-sectional associations, and expert opinion; none have been prospectively validated as triggers for clinical intervention. Second, the relationship between improving TITR and long-term cardiovascular or mortality outcomes is inferred from observational data and post-hoc analyses, and causality remains unproven. Third, different CGM platforms exhibit slight variations in accuracy and data handling, which may influence the generalisability of specific metric cut-points. Fourth, the framework currently lacks integration with cost-effectiveness analyses and provides limited guidance for settings with severely restricted CGM access. Fifth, although we advocate for sustainable stability, the optimal trade-off between tight glycaemic control and patient quality of life has not been empirically defined. These uncertainties underscore the urgent need for the validation studies outlined above.

## Conclusion

By embedding TITR within disease classification, the 2026 staging proposal elevates CGM from a management adjunct to a diagnostic and prognostic tool of genuine clinical utility. Its ultimate impact, however, hinges on closing the CGM decision gap—the persistent disconnect between the granularity of glycaemic data and the specificity of therapeutic response. The practical three-step closed-loop model proposed here, anchored in the principle of sustainable glycaemic stability and tailored to each disease stage, offers a conceptual roadmap to bridge data and durable action. CGM has already proven its capacity to visualise dysglycaemia with unprecedented resolution. The immediate task is to ensure it can also guide—in a language that both clinicians can act upon and patients can sustain—toward reduced lifelong complication risk. Achieving this vision will require not only prospective validation of the framework in diverse populations, but also systematic attention to implementation barriers, cost-effectiveness, and the integration of patient-reported outcomes to ensure that the pursuit of metabolic stability remains aligned with quality of life.

## Data Availability

The original contributions presented in the study are included in the article/supplementary material. Further inquiries can be directed to the corresponding author.
